# Primary care physician digital health profile and burnout: an international cross-sectional study

**DOI:** 10.1093/eurpub/ckaf106

**Published:** 2025-07-15

**Authors:** Mathieu Jendly, Valérie Santschi, Stefano Tancredi, Viktor von Wyl, Arnaud Chiolero

**Affiliations:** Population Health Laboratory (#PopHealthLab), University of Fribourg, Fribourg, Switzerland; La Source, School of Nursing Sciences, HES-SO University of Applied Sciences and Arts Western Switzerland, Lausanne, Switzerland; Population Health Laboratory (#PopHealthLab), University of Fribourg, Fribourg, Switzerland; Swiss School of Public Health (SSPH+), Zurich, Switzerland; Swiss School of Public Health (SSPH+), Zurich, Switzerland; Epidemiology, Biostatistics and Prevention Institute – EBPI, University of Zurich, Zurich, Switzerland; Institute for Implementation Science in Health Care – IfIS, University of Zurich, Zurich, Switzerland; Population Health Laboratory (#PopHealthLab), University of Fribourg, Fribourg, Switzerland; Swiss School of Public Health (SSPH+), Zurich, Switzerland; School of Population and Global Health, McGill University, Montreal, QC, Canada; Institute of Primary Health Care (BIHAM), University of Bern, Bern, Switzerland

## Abstract

**Background:**

Digital health offers promising solutions for enhancing patient care, yet adoption varies among physicians, partly due to concerns about administrative burdens and burnout. This study assessed digital health use and burnout among primary care physicians in 10 OECD countries and examined their relationship.

**Methods:**

We conducted a secondary analysis of “The Commonwealth Fund’s 2022 International Health Policy Survey,” including 9526 randomly selected primary care physicians (general practitioners or pediatricians in ambulatory care) from 10 OECD countries. We created a digital health score based on the use and frequency of digital tools. Self-reported burnout and related outcomes were analyzed. Cross-country differences were assessed using stratified analyses. Associations between digital health and burnout and related outcomes were explored using stratified analyses and logistic regressions.

**Results:**

Most physicians used electronic records; video consultations or connected tools for chronic care. Digital health scores were highest in the Netherlands and UK, and lowest in Germany and Switzerland. 35% of physicians reported burnout, with the highest prevalence in New Zealand (49%) and Canada (46%), and lowest in the Netherlands (12%) and Switzerland (18%). Digital health use positively correlated with workload dissatisfaction but not with burnout, stress, satisfaction with administrative work, or work-life balance.

**Conclusion:**

Physicians’ digital health use and burnout varied substantially across countries but were not correlated. While digital health is often considered a factor linked to physician burnout, our results do not support this view. They also highlight the need to ensure that digital health reduces, rather than exacerbates, physicians’ workload.

## Introduction

Digital health encompasses technologies such as mobile health (mHealth), health information technology, wearable devices, telehealth, and telemedicine [1]. It holds promise for improving chronic conditions management [2, 3] and enhancing patient well-being [4]. For instance, digital health enables patient-centred health services through communication technologies [5, 6], which are increasingly important amid the growing burden of chronic diseases in aging populations [7]. Despite its promises, the adoption of digital health faces significant challenges at both individual and systemic levels. For example, physicians often worry about disruptions to patient relationships [8] and insufficient evidence on the benefits and risks of digital tools [9]. The adoption of digital tools is further complicated by disparities in their access across countries [10]. Primary care physicians are central to this adoption, as they are often the first point of contact in the healthcare system. Hence, they could play a key role in guiding patients in the use of such technologies [11].

The Unified Theory of Acceptance and Use of Technology (UTAUT) framework helps explain barriers to the implementation of digital health tools. Within this framework, four main factors—performance expectancy, effort expectancy, social influence, and facilitating conditions—determine whether individuals adopt or resist new technologies [12, 13]. Concerns about the effectiveness of digital tools may lower performance expectancy. Increased administrative burden caused by digital health tools [14] may lower effort expectancy, as physicians may perceive these tools as too demanding. While one of their goals is to reduce the workload, they can also paradoxically contribute to burnout, an effect that could be mitigated through improved technology and workflow optimization [15]. These dynamics help understand healthcare professionals’ resistance to adoption.

Burnout is also a factor that may be intertwined with the adoption of digital health. Defined as “a syndrome […] resulting from chronic workplace stress that has not been successfully managed” [16], it is frequent among physicians [17]. The Commonwealth Fund’s 2022 International Health Policy Survey found that nearly half of primary care physicians in several OECD (Organisation for Economic Co-operation and Development) countries reported being burned out, which could accelerate the primary care shortage [18]. Burnout correlates with reduced care quality and empathy, and increased clinical errors [19]. On the one hand, the growing administrative burden associated with electronic health records is a recognized contributor to burnout [20]. On the other hand, digital health could also act as a facilitator, due to its ability to alleviate some of these burdens [21], potentially giving physicians more time for clinical activities and patient interaction. Understanding the link between primary care physician burnout and digital health adoption is important, as primary care physicians play a pivotal role in the wider uptake of digital health technologies by patients.

Therefore, using data from a large survey of primary care physicians across 10 OECD countries, we aimed to assess cross-country differences in primary care physician digital health use and burnout, as well as the relationship between digital health use and burnout.

## Methods

### Study design

We conducted a secondary analysis of the 2022 International Health Policy (IHP) Survey of the Commonwealth Fund (CWF), whose methodology has been described elsewhere [22]. The CWF is a non-profit foundation in the USA that has been conducting IHP surveys since 1998 to compare the health system performances in USA and several other high-income countries. Three target groups are surveyed every three years, that is, the resident population aged 18 years and over, the resident population aged 65 years and over, and primary care physicians [23].

Our analysis focused on the 2022 IHP physician survey conducted in 10 OECD countries (Australia, Canada, France, Germany, the Netherlands, New Zealand, Sweden, Switzerland, the United Kingdom, and the United States of America). Ethical approval was obtained for the original survey, no additional approval was required for secondary analysis.

### Target and study populations

We included all respondents to the 2022 IHP survey. The target populations are primary care physicians not working in hospitals. In several countries (e.g. Australia, New Zealand, the Netherlands, and the UK) included in this survey, primary care physicians treat children and adults. In countries where primary care physicians exclusively treat adults (the United States of America and Switzerland), pediatricians working in ambulatory care were also invited to participate and represented 14% and 16% of physicians, respectively. Germany excluded pediatricians from the sample. The CWF partnered with various statistics companies to identify the target population (i.e. all potentially eligible physicians) and obtained a random sample from this group to send the questionnaire. However, the number of physicians in the target population (i.e. number of potentially eligible physicians per country) was not specified for some countries. The eligibility of participants was assessed by the local agencies that provided lists of participants. Ineligible participants were mainly composed of primary care physicians who screened out as not being involved in primary care, being retired, having changed addresses or phone numbers, and being deceased. In Germany, survey invitations were managed by the regional medical associations, with varying approaches: five out of seventeen associations contacted all members by email, nine included a survey note in their newsletter, and three did not disclose their recruitment strategy. Consequently, a response rate could not be calculated for Germany, and the detailed participation process could not be precisely described [22].

The targeted study populations, the random sample of physicians eligible to participate, the sample of physicians invited to participate, and the number of participants per country are shown in Supplementary Table S1. In total, 9526 completed the questionnaire and constituted our study sample (Supplementary Fig. S6), with participation ranging from 4.3% to 39.1% across countries.

### Data collection and measurement

Between February and September 2022, physicians completed an online, mail, postal, or phone questionnaire covering personal and workplace characteristics, their work-related satisfaction, their burnout symptoms, and digital health use. For our analysis, we used the following physician and workplace characteristics to provide insights into the type of medical practice across countries: country, age, gender, degree of urbanization of the practice location, weekly working hours, number of patients seen per week, and number of full-time equivalents in the practice. These characteristics are displayed in Table 1.

**Table 1.** Characteristics of primary care physicians and practice care settings (*N* = 9526)

Characteristics	*N* (%)
Country	
Australia	321 (3)
Canada	1459 (15)
France	530 (6)
Germany	947 (10)
Netherlands	617 (6)
New Zealand	377 (4)
Sweden	2092 (22)
Switzerland	1114 (12)
United Kingdom	1010 (11)
United States	1059 (11)
Age [year]	
Under 35	1041 (11)
35–44	2755 (29)
45–54	2330 (24)
55–64	2287 (24)
65 or older	1097 (12)
Gender	
Women	4573 (48)
Men	4910 (52)
Other	11 (<1)
Community type	
Urban	4065 (43)
Intermediate	3525 (37)
Rural	1901 (20)
Weekly working hours	
Less than 35	2188 (23)
35–44	2654 (28)
45 or more	4500 (47)
Number of patients seen per week	
Less than 70	3771 (40)
70–119	2721 (29)
120 or more	2712 (28)
Number of patients seen per hour	
Less than 2	4519 (47)
2–4	3521 (37)
4 or more	954 (10)
Number of full-time equivalents in the office	
Less than 2	2191 (23)
2–3	1392 (15)
3–6	2923 (31)
6 or more	2681 (28)

Physicians’ digital health use was assessed by computing a score based on responses to 10 questions about teleconsultation, use of connected health tools, electronic patient records, and various online services for patients. Table 2 shows the items on which the digital health score is based, by assigning a value of 0 or 1 to each response, and by adding up these values to get a score ranging from 0 to 10. The higher the digital health score, the higher the physicians’ use and involvement in digital health. The score was created based on background knowledge and authors’ expertise [24] because it is simple yet comprehensive, covering many aspects of digital health, such as telemedicine, connected health tools, electronic health records, and various aspects of digital services among doctors and between patients and practices. Furthermore, no previous score that assessed physicians’ digital health use based on these survey data could be found. This score was used in our previous research using the same dataset to assess the digital health profile of primary care physicians in Switzerland [24].

**Table 2.** Digital health score items and burnout and related outcomes of primary care physicians (*N* = 9526)

	Variable	Outcome [score^a^]	*N* (%)
Digital health use	Percentage of consultations by video	5% or more [1]	2325 (24)
		Less than 5% [0]	7079 (74)
	Use of connected health tools to monitor the health of patients with chronic diseases	25% or more [1]	3529 (37)
		Less than 25% [0]	5796 (61)
	Use of electronic patient medical records	Yes [1]	8812 (93)
		No [0]	680 (7)
	Possibility to communicate electronically patient clinical summaries	Yes [1]	5947 (62)
		No [0]	3286 (35)
	Possibility to communicate electronically diagnostic and laboratory tests	Yes [1]	6560 (69)
		No [0]	2690 (28)
	Possibility to communicate electronically list of medications	Yes [1]	6109 (64)
		No [0]	3089 (32)
	Practice allowing e-mail or web communications with patients	Yes [1]	7216 (76)
		No [0]	2181 (23)
	Practice allowing online appointment taking with patients	Yes [1]	4854 (51)
		No [0]	4367 (46)
	Practice allowing online medical prescriptions renewal	Yes [1]	5691 (60)
		No [0]	3728 (39)
	Practice allowing online lab results acknowledgement by patients	Yes [1]	5424 (57)
		No [0]	3885 (41)
Burnout self-assessment and burnout-related outcomes	Overall, based on your definition of burnout, how would you rate your current level of burnout?	I enjoy my work. I have no symptoms of burnout.	1286 (14)
		Occasionally I am under stress, and I don’t always have as much energy as I once did, but I don’t feel burned out.	4807 (50)
		I am definitely burning out and have one or more symptoms of burnout, such as physical and emotional exhaustion.	2193 (23)
		The symptoms of burnout that I’m experiencing won’t go away. I think about frustration at work a lot.	736 (8)
		I feel completely burned out and often wonder if I can go on. I am at the point where I may need some changes or may need to seek some sort of help.	439 (5)
	How stressful is your job?	Extremely stressful	1927 (20)
		Very stressful	3666 (38)
		Somewhat stressful	3228 (34)
		Not too stressful	553 (6)
		Not stressful at all	81 (1)
	Satisfaction with daily workload	Extremely satisfied	123 (1)
		Very satisfied	1030 (11)
		Moderately satisfied	2943 (31)
		Slightly satisfied	2875 (30)
		Not at all satisfied	2518 (26)
	Satisfaction with time spent on administrative work	Extremely satisfied	92 (1)
		Very satisfied	319 (3)
		Moderately satisfied	1431 (15)
		Slightly satisfied	2890 (30)
		Not at all satisfied	4754 (50)
	Satisfaction with work-life balance	Extremely satisfied	302 (3)
		Very satisfied	1400 (15)
		Moderately satisfied	3339 (35)
		Slightly satisfied	2604 (27)
		Not at all satisfied	1830 (19)

a Values used to build the digital health score. We summed all variables to obtain a score ranging from 0 to 10.

Burnout was assessed using a single categorical self-reported item, as shown in Table 2. Physicians were considered in burnout if their answer to the question was: “I am burning out and have one or more symptoms of burnout, such as physical and emotional exhaustion,” “The symptoms of burnout that I’m experiencing won’t go away. I think about frustration at work a lot,” or “I feel completely burned out and often wonder if I can go on. I am at the point where I may need some changes or may need to seek some sort of help.” On the one hand, this single-item measure was deemed appropriate, given that physicians are familiar with burnout through both their own experiences and their clinical practice, which should render them relatively well-equipped to provide reliable self-assessments, at least when the burnout is severe. On the other hand, one major problem is that the way physicians perceive and diagnose burnout differs certainly from one country to the other, and even from one physician to the other. We also assessed several burnout-related variables such as perceived stress, satisfaction with daily workload, time spent on administrative tasks, and overall satisfaction with work-life balance.

### Statistical analysis

First, we presented descriptive statistics for physician characteristics, digital health use, digital health score, and their burnout and related outcomes, i.e. perceived stress, satisfaction with daily workload, time spent on administrative tasks, and with work-life balance. Categorical variables are displayed as numbers and percentages, and continuous variables are displayed as mean and standard deviation.

Second, we conducted stratified analyses to assess cross-country differences in digital health scores and burnout levels. Three models were used to compute adjusted estimates of the digital health score and burnout rankings, shown in Supplementary Table S3. Model 1 shows the unadjusted means and prevalence, respectively. Model 2 was adjusted for age and gender, and model 3 was adjusted for age, gender, community type, weekly working hours, number of patients seen per week and per hour, and number of full-time equivalents in the practice. The adjustments were calculated using the standard post-estimation command “margins.”

Third, we used stratified analyses and logistic regressions to examine the association between the digital health score and both burnout and related outcomes. We performed unadjusted analyses, then adjusted for age, gender, and country. Age was included because it could influence both the adoption of digital health tools and burnout, with younger physicians possibly more prone to using technology but experiencing higher stress. Gender was adjusted because it has been shown to be associated with digital health use [25] and burnout [26]. We adjusted for country to address differences in healthcare infrastructure and digital health implementation that could influence both digital health use and burnout.

For each variable, the number and percentage of missing data and alternative responses, such as “declined to answer” or “not sure” are detailed in Supplementary Table S4. Only a few data were missing or marked as alternative responses. For the digital health score items, a value of 0 was assigned if data was missing, if there was an inaccessible multiple response code, or if the participant was unsure or declined to answer.

### Data availability

Data are publicly available by contacting the CWF or local health agencies of the participating countries. The code used to perform the analyses of this study can be shared upon request.

### Writing and editing assistance

Language editing assistance for improving clarity and fluency of the English manuscript was provided by a large language model (ChatGPT^®^ by OpenAI).

## Results

The characteristics of the participants and their care practice setting are shown in Table 1. A total of 9526 primary care physicians from 10 OECD countries participated. Most were aged between 35 and 64 years (77%) and 48% were women. 43% practiced in urban, 37% in intermediate, and 20% in rural settings. Some 47% reported working 45 or more hours per week. Most worked together with 3 or more full-time equivalents per practice. Despite efforts to ensure comparability through random sampling, participant characteristics varied between countries (Supplementary Table S2). Physicians in Canada, France, Sweden, and the UK were generally younger. Physicians from Germany and the Netherlands were more likely to work in intermediate or rural settings. Participants from Australia and Germany reported the highest numbers of consultations per week and per hour. Solo or small-team practices (fewer than three full-time equivalents) were more common in France, Germany, the Netherlands, and Switzerland.

Table 2 shows the digital health score items, burnout and burnout-related outcomes. Electronic medical records were the most used tool (93%). Some 24% used video consultation with at least 5% of their patients, and 37% were using connected health tools (for instance connected blood glucose meters or smartwatches) to monitor chronic conditions with at least 25% of their patients. Some 23% of the participants reported symptoms of burnout, 8% answered that these symptoms would not go away, and 5% stated they were completely burned out and often wondered if they could go on the way they do. Job stress was reported as very or extremely high by 58%. Dissatisfaction was reported by 26% for daily workload, 50% for administrative tasks, and 19% for work-life balance.

**Figure 1.**
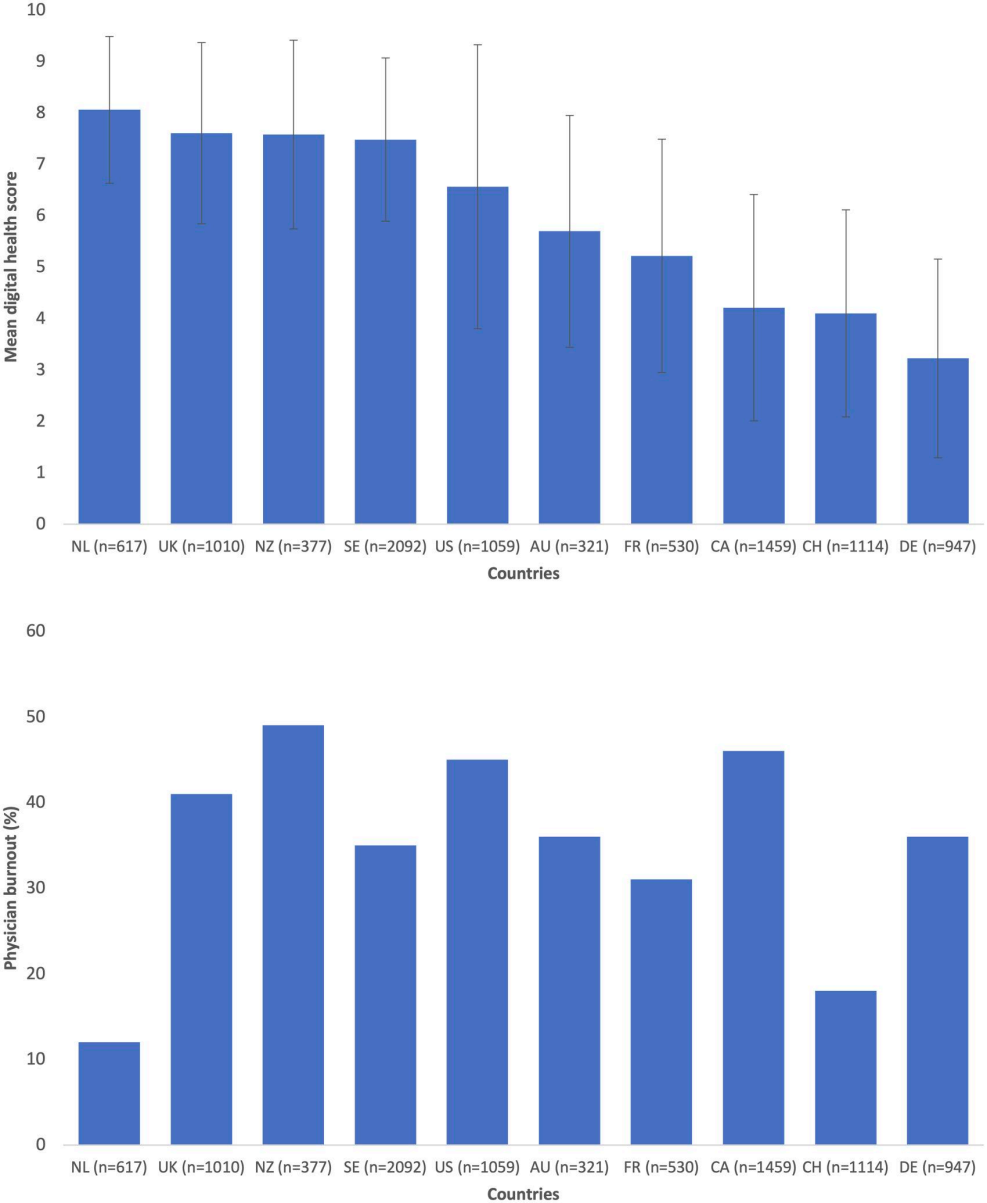
Digital health score and physician burnout by country (Country codes: NL = Netherlands, UK = United Kingdom, NZ = New Zealand, SE = Sweden, US = United States, AU = Australia, FR = France, CA = Canada, CH = Switzerland, DE = Germany). The digital health score is described by mean and standard deviation, and physician burnout is described as percentage of prevalence (*N* = 9526).

Figure 1 shows the cross-country differences in the average digital health score, ranging from 0 to 10, as well as in burnout rates. The Netherlands had the highest mean score (8.1, SD: 1.4), followed by the United Kingdom (7.6, SD: 1.8) and New Zealand (7.6, SD: 1.8). Switzerland (4.1, SD: 2.0) and Germany (3.2, SD: 1.9) had the lowest mean score. Supplementary Figure S7 shows the substantial cross-country differences in burnout self-assessment and burnout-related outcomes. Burnout rates were highest in New Zealand (49%), Canada (46%) and the United States (45%), and lowest in Switzerland (18%) and the Netherlands (12%). Job stress shows high cross-country differences with the highest perceived stress in Germany (73%) and the United States (72%), and the lowest stress rates were found in Switzerland (43%) and the Netherlands (32%). Participants from UK, New Zealand, the Netherlands and Canada reported the most dissatisfaction across workload, administrative work and work-life balance. Supplementary Table S3 shows the three models used to adjust for differences in physician characteristics between countries. Adjusting for age and gender (Model 2), and for age, gender, community type, weekly working hours, number of patients seen per week and per hour, and number of full-time equivalents in the practice (Model 3) did not change the rankings in digital health score and in burnout prevalence.

**Figure 2.**
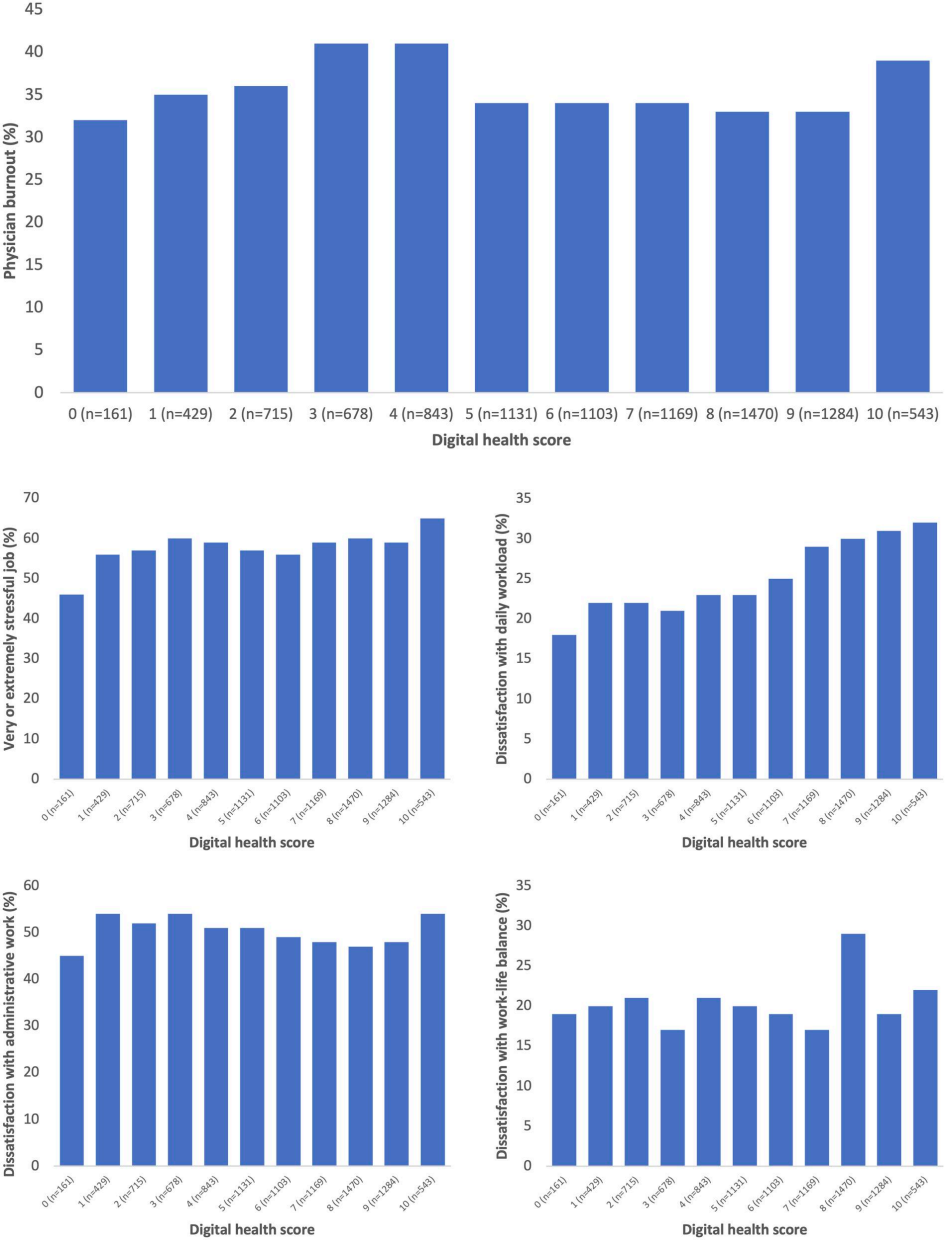
Proportion of physician reporting burnout and burnout-related outcomes by digital health score (*N* = 9526).

Figure 2 presents unadjusted stratified analyses between digital health score and burnout and related outcomes. No association was apparent between the digital health score and burnout, perceived work stress, dissatisfaction with time spent on administrative tasks, and dissatisfaction with work-life balance. However, higher digital health scores were linked to greater dissatisfaction with daily workload. This was confirmed by logistic regression (Supplementary Table S5) adjusted for age, gender and country, showing that dissatisfaction with workload was positively associated with the digital health score (OR 1.08 [1.06–1.10]). No associations were found with burnout (OR 0.99), perceived work stress (OR 1.02), dissatisfaction with time spent on administrative tasks (OR 0.99), or dissatisfaction with work-life balance (OR 1.00).

## Discussion

The aim of this study was to assess cross-country differences in primary care physician digital health use and burnout and their relationship, using the results of large surveys in 10 OCDE countries. The Netherlands, the United Kingdom, and New Zealand had the highest level of digital health adoption, Switzerland and Germany the lowest. Burnout was most frequent in New Zealand, Canada, and the United States (about 50%), and least common in Switzerland and the Netherlands. We did not find any association between the digital health score and burnout.

Our findings indicate a positive association between dissatisfaction with daily workload and digital health use. This may suggest that while digital health tools are intended to support certain clinical tasks, they could also contribute to a higher workload. For instance, the increased administrative burden associated with use of electronic health records and other digital systems may demand high time spent on documentation, leading to frustration and dissatisfaction among physicians [27]. On the other hand, it is also possible that physicians with heavier workloads are more likely to adopt digital health tools to cope with the demands of their practice [28]. While this dual interpretation of these findings is plausible, it remains speculative, as our cross-sectional data do not allow us to assess the directionality or causality of this association. This underscores the need to further investigate how digital health can be optimized to reduce workload without adding additional stress.

Our findings align with previous research. The Bertelsmann Stiftung, an independent foundation that seeks to promote social change, published a ranking identifying the Netherlands, the United Kingdom, and New Zealand as leaders in digital health policy activity and actual use of data [29]. It also identified a combination of structural, institutional, and policy-related factors—including legal frameworks, financing, and the presence of national coordinating agencies—as key contributors to successful digital health implementation. Countries with a stronger institutional anchoring, such as Denmark and the Netherlands, demonstrated higher levels of digital health use. As in the case of Switzerland, the authors suggest that the use of digital health data “is rather low, which is due to the relatively recent implementation of the EPD (Electronic Patient Dossier) law.” For low-performing countries to achieve a better adoption and utilization of digital health, this report suggests key factors for a successful implementation: establishing a digital health institution with coordinating power; assuring cooperation among stakeholders; and involving end-users like physicians and patients in the development of tools [30]. Multiple studies have shown that electronic health records, while intended to increase efficiency, may increase administrative burdens and contribute to higher levels of stress among physicians when poorly designed. For example, research has demonstrated that increased time spent on electronic health record tasks is associated with higher levels of burnout and stress [27].

Interpreted through the lens of the UTAUT framework, these results contribute to further hypotheses about the barriers and facilitators affecting digital health adoption among primary care physicians. In terms of performance expectancy, physicians in the countries with high adoption might expect digital tools to enhance their performance by reducing clinical errors or improving patient management. Regarding effort expectancy, our findings show that higher digital health use is associated with lower satisfaction with daily workload. Physicians may find the effort required to use these tools outweighs the perceived benefits, which aligns with the increased dissatisfaction observed. Social influence may also impact adoption, and countries with higher adoption may have stronger cultural and professional norms supporting technology use in healthcare. Finally, facilitating conditions, such as better infrastructure, training, and policy support, are essential for effective adoption.

Our study has several limitations. First, it is a secondary analysis from data not designed to study the relationship between digital health use and burnout. The survey was completed partly online, and the 18% participation rate (excluding Germany) raises concerns about participation bias, potentially favoring digitally engaged physicians and underrepresenting those with severe burnout. Second, sampling and participation rates varied across country, potentially introducing a participation bias that differed from one country to the other. Participant characteristics were heterogenous. Third, the digital health score used, while comprehensive and used in a previous study [24], is neither weighted nor validated and may not capture digital health use entirely, such as the quality of the tools used or their integration into clinical workflows. Furthermore, this score mixes different services, and some of its aspects could have opposite effects on the workload of primary care physicians, and, eventually, burnout. For instance, tools such as online appointment scheduling, or electronic prescription renewal may streamline workflows and reduce administrative burden, while other tools like e-mail or web messaging with patients or remote monitoring of chronic conditions may increase workload [31]. Future research should explore this heterogeneity further, potentially by disaggregating the digital health score to better understand the specific associations of its components with physician burnout. Fourth, burnout was assessed with a single self-reported item and did not use more comprehensive tools, such as the Maslach Burnout Inventory (MBI) [32]. As a result, it did not capture the multidimensional and complex nature of burnout [33]. Nevertheless, the burnout rates reported in this study are similar to those reported in other studies, for instance in the USA [34], Canada [35], and in Germany [36]. Fifth, we could not consider in the analysis other potentially important determinants of digital tools use such as level of training, or institutional implementation strategies. Last, we cannot assume a causal relationship between the analyzed variables. Future research should use longitudinal or experimental designs to better assess the directionality of these associations and explore causal pathways and identify interventions easing the implementation of digital tools without increasing the burden of physicians.

In conclusion, our study revealed massive cross-country differences in digital health use and in burnout rates. Burnout and digital health use were not associated. However, the negative relationship between digital health use and physician workload satisfaction might imply that digital health may add to the administrative burden for physicians. If true, this indicates the need for optimization of digital health tools to ensure that they reduce, rather than exacerbate, physicians’ workload. Our results suggest that implementation should focus on aligning these tools with physicians’ workflows. Policymakers, patients, and healthcare professionals involved in the co-design of such tools may use these results to prioritize strategies that help administrative processes and invest in infrastructures that support user-friendly digital systems. Technology developers could benefit from insights into which features impact workload satisfaction to improve adoption of their tools. These insights can help guide targeted implementation strategies to ensure digital health fulfills its promises without compromising physician well-being.

## Author contributions

M.J., V.S., S.T., V.v.W., and A.C. wrote the protocol for the current study based on an idea of M.J., V.S., and A.C. M.J. analyzed the data under the guidance of A.C. and drafted the manuscript with contributions of V.S., S.T., V.v.W., and A.C. All authors revised and approved the final version of the manuscript before submission.

## Supplementary Material

ckaf106_Supplementary_Data
