# Supplementary material for: Primary care physician digital health profile and burnout: an international cross-sectional study
Source: Eur J Public Health. 2025 Jul 15;35(6):1162–9. doi: 10.1093/eurpub/ckaf106 (PMC12707498; doi:10.1093/eurpub/ckaf106)
Supplement: ckaf106_Supplementary_Data [file ckaf106_supplementary_data.docx]

Primary care physician eHealth profile and burnout: an international cross-sectional study

Mathieu Jendly^1^, Prof. Valérie Santschi^2^, Dr. Stefano Tancredi^1,3^, Prof. Viktor von Wyl^3,4,5^, Prof. Arnaud Chiolero^1,3,6,7*^

^1^ Population health laboratory (#PopHealthLab), University of Fribourg, Fribourg, Switzerland

^2^ La Source, School of Nursing Sciences, HES-SO University of Applied Sciences and Arts Western Switzerland, Lausanne, Switzerland

^3^ Swiss School of Public Health (SSPH+), Zurich

^4^ Epidemiology, Biostatistics and Prevention Institute - EBPI, University of Zurich, CH-8001 Zurich, Switzerland

^5^ Institute for Implementation Science in Health Care- IfIS, University of Zurich, CH-8001 Zurich, Switzerland

^6^ School of Population and Global Health, McGill University, Montreal, QC, Canada

^7^ Institute of Primary Health Care (BIHAM), University of Bern, Bern, Switzerland

*** Correspondence:**Prof Arnaud Chiolero, MD, PhD
Population health laboratory
University of Fribourg
Route des Arsenaux 41
1700 Fribourg
[arnaud.chiolero@unifr.ch](mailto:arnaud.chiolero@unifr.ch)

**Appendix**

**Supplementary Table S1**: Target population, random sample, physicians invited to participate, participants (N total = 9526), and participation rate by country. Some values were not available (*NA*).

| Country | Target population^a^ | Random sample^b^ | Non-deliverables and ineligibles^c^ | Physicians invited to participate^d^ | Participants^e^ | Participation rate^f^ |
| --- | --- | --- | --- | --- | --- | --- |
| Australia | 25000 | 3502 | 492 | 3010 | 321 | 10,7% |
| Canada | 90000 | 6478 | 12 | 6466 | 1459 | 22,6% |
| France | 68196 | 12650 | 487 | 12163 | 530 | 4,3% |
| Germany | 26396 | NA | NA | NA | 947 | NA |
| Netherlands | 4800 | 1600 | 20 | 1580 | 617 | 39,1% |
| New Zealand | NA | 3936 | 17 | 3919 | 377 | 9,6% |
| Sweden | NA | 6000 | 15 | 5985 | 2092 | 35,0% |
| Switzerland | 8354 | 4000 | 59 | 3941 | 1114 | 28,3% |
| United Kingdom | 72722 | 4606 | 20 | 4586 | 1010 | 22,0% |
| United States | 350000 | 5852 | 53 | 5799 | 1059 | 18,3% |

a: (approximate) number of potentially eligible physicians per country.

b: random sample of physicians eligible for participation from the study population provided by local health agencies.

c: participants excluded from the random sample.

d: sample after exclusion of non-deliverables and ineligibles.

e: sample who completed the questionnaire.

f: participants divided by the valid sample.

**Supplementary Table S2**: Characteristics of primary care physicians and practice care settings by country (N = 9526).

|  |  | N (%) | | | | | | | | | |
| --- | --- | --- | --- | --- | --- | --- | --- | --- | --- | --- | --- |
| Characteristics |  | Australia (n=321) | Canada (n=1459) | France (n=530) | Germany (n=947) | Netherlands (n=617) | New Zealand (n=377) | Sweden (n=2092) | Switzerland (n=1114) | United Kingdom (n=1010) | United States (n=1059) |
| Age [year] | Under 35 | 14 (4) | 259 (18) | 102 (19) | 22 (2) | 27 (4) | 17 (5) | 289 (14) | 33 (3) | 226 (22) | 52 (5) |
|  | 35-44 | 54 (17) | 355 (24) | 163 (31) | 179 (19) | 181 (29) | 70 (19) | 767 (37) | 278 (25) | 505 (50) | 203 (19) |
|  | 45-54 | 89 (28) | 324 (22) | 85 (16) | 312 (33) | 182 (30) | 92 (24) | 475 (23) | 333 (30) | 168 (17) | 270 (26) |
|  | 55-64 | 107 (33) | 316 (22) | 141 (27) | 340 (36) | 196 (32) | 144 (38) | 351 (17) | 318 (29) | 87 (9) | 287 (27) |
|  | 65 or older | 57 (18) | 197 (14) | 39 (7) | 94 (10) | 29 (5) | 54 (14) | 210 (10) | 152 (14) | 24 (2) | 241 (23) |
| Gender | Women | 138 (43) | 763 (52) | 265 (50) | 457 (48) | 298 (48) | 218 (57) | 1198 (57) | 518 (47) | 607 (60) | 448 (42) |
|  | Men | 181 (56) | 688 (47) | 261 (49) | 490 (52) | 314 (51) | 158 (42) | 894 (43) | 596 (54) | 401 (40) | 590 (56) |
|  | Other | 2 (1) | 5 (<1) | 0 (0) | 0 (0) | 0 (0) | 1 (<1) | 0 (0) | 0 (0) | 2 (<1) | 1 (<1) |
| Community type | Urban | 196 (61) | 682 (47) | 278 (52) | 246 (26) | 213 (35) | 205 (54) | 818 (39) | 466 (42) | 523 (52) | 438 (41) |
|  | Intermediate | 95 (30) | 376 (26) | 62 (12) | 431 (46) | 320 (52) | 125 (33) | 835 (40) | 404 (36) | 410 (41) | 467 (44) |
|  | Rural | 30 (9) | 392 (27) | 190 (36) | 268 (28) | 77 (12) | 46 (12) | 439 (21) | 243 (22) | 72 (7) | 144 (14) |
| Weekly working hours | Less than 35 | 129 (40) | 214 (15) | 81 (15) | 58 (6) | 68 (11) | 159 (42) | 640 (31) | 287 (26) | 406 (40) | 146 (14) |
|  | 35-44 | 94 (29) | 310 (21) | 154 (29) | 127 (13) | 159 (26) | 107 (28) | 880 (42) | 258 (23) | 321 (32) | 244 (23) |
|  | 45 or more | 96 (30) | 843 (58) | 291 (55) | 758 (80) | 389 (63) | 110 (29) | 545 (26) | 568 (51) | 282 (28) | 618 (58) |
| Number of patients seen per week | Less than 70 | 62 (19) | 421 (29) | 58 (11) | 27 (3) | 110 (18) | 178 (47) | 1789 (86) | 441 (40) | 232 (23) | 453 (43) |
|  | 70-119 | 86 (27) | 489 (34) | 234 (44) | 95 (10) | 233 (38) | 142 (38) | 232 (11) | 418 (38) | 384 (38) | 408 (39) |
|  | 120 or more | 171 (53) | 444 (30) | 228 (43) | 769 (81) | 264 (43) | 56 (15) | 0 (0) | 251 (23) | 390 (39) | 139 (13) |
| Number of patients seen per hour | Less than 2 | 46 (14) | 637 (44) | 90 (17) | 66 (7) | 182 (30) | 170 (45) | 1813 (87) | 638 (57) | 169 (17) | 708 (67) |
|  | 2-4 | 170 (53) | 562 (39) | 387 (73) | 341 (36) | 384 (62) | 195 (52) | 123 (6) | 442 (40) | 681 (67) | 236 (22) |
|  | 4 or more | 100 (31) | 99 (7) | 40 (8) | 444 (47) | 41 (7) | 10 (3) | 11 (1) | 29 (3) | 154 (15) | 26 (2) |
| Number of full-time equivalents in the office | Less than 2 | 28 (12) | 277 (19) | 216 (41) | 314 (33) | 367 (59) | 55 (15) | 58 (3) | 550 (49) | 42 (4) | 274 (26) |
|  | 2 to 3 | 21 (7) | 193 (13) | 118 (22) | 238 (25) | 121 (20) | 63 (17) | 127 (6) | 268 (24) | 98 (10) | 145 (14) |
|  | 3 to 6 | 127 (40) | 418 (29) | 173 (33) | 210 (22) | 114 (18) | 157 (42) | 723 (35) | 214 (19) | 505 (50) | 282 (27) |
|  | 6 or more | 153 (42) | 529 (36) | 23 (4) | 44 (5) | 11 (2) | 102 (27) | 1081 (52) | 78 (7) | 351 (35) | 327 (31) |

**Supplementary Table S3**: Digital health score and physician burnout by country*. The digital health score is described by mean and standard deviation, and physician burnout is described as percentage of prevalence (N = 9526).

|  |  | Means (SD) | Margins (SD) | |
| --- | --- | --- | --- | --- |
|  | Countries | Model 1 ** | Model 2 ** | Model 3 ** |
| Digital health score | NL (n=617) | 8.06 (1.43) | 8.06 (0.81) | 8.02 (0.82) |
|  | UK (n=1010) | 7.61 (1.77) | 7.61 (0.06) | 7.60 (0.06) |
|  | NZ (n=377) | 7.58 (1.84) | 7.58 (0.10) | 7.58 (0.10) |
|  | SE (n=2092) | 7.48 (1.59) | 7.48 (0.04) | 7.51 (0.04) |
|  | US (n=1059) | 6.56 (2.76) | 6.56 (0.06) | 6.54 (0.06) |
|  | AU (n=321) | 5.70 (2.25) | 5.70 (0.11) | 5.68 (0.11) |
|  | FR (n=530) | 5.22 (2.27) | 5.22 (0.09) | 5.20 (0.09) |
|  | CA (n=1459) | 4.21 (2.20) | 4.22 (0.05) | 4.22 (0.05) |
|  | CH (n=1114) | 4.10 (2.01) | 4.10 (0.06) | 4.08 (0.06) |
|  | DE (n=947) | 3.22 (1.93) | 3.22 (0.07) | 3.23 (0.07) |
|  |  | Prevalence | Margins (SD) | |
| Burnout | NL (n=617) | 12 | 12 (1) | 11 (1) |
|  | UK (n=1010) | 41 | 41 (2) | 44 (2) |
|  | NZ (n=377) | 49 | 49 (3) | 52 (3) |
|  | SE (n=2092) | 35 | 34 (1) | 37 (1) |
|  | US (n=1059) | 45 | 46 (2) | 44 (2) |
|  | AU (n=321) | 36 | 36 (3) | 40 (3) |
|  | FR (n=530) | 31 | 31 (2) | 30 (2) |
|  | CA (n=1459) | 46 | 46 (3) | 44 (1) |
|  | CH (n=1114) | 18 | 18 (1) | 19 (1) |
|  | DE (n=947) | 36 | 36 (2) | 32 (2) |

* Country codes: NL = Netherlands, UK = United Kingdom, NZ = New Zealand, SE = Sweden, US = United States, AU = Australia, FR = France, CA = Canada, CH = Switzerland, DE = Germany

* Model 1 was unadjusted, Model 2 was adjusted for age and gender, and model 3 was adjusted for age, gender, community type, weekly working hours, number of patients seen per week and per hour, and number of full-time equivalents in the practice.

**Supplementary Table S4**: Number of missing and alternative data for each variable (N = 9526). Some data were not available (NA).

| Variable | Missing | Invalid | Multiple-response code | Not sure | Prefer not to answer | Declined to answer |
| --- | --- | --- | --- | --- | --- | --- |
| Country | 0 (0) | *NA* | *NA* | *NA* | *NA* | *NA* |
| Age [year] | 0 (0) | *NA* | 0 (0) | 0 (0) | *NA* | 16 (0) |
| Gender | 0 (0) | *NA* | 0 (0) | 0 (0) | 10 (0) | 22 (0) |
| Community type | 0 (0) | *NA* | 16 (0) | 0 (0) | *NA* | 19 (0) |
| Weekly working hours | 0 (0) | 112 (1) | *NA* | *NA* | *NA* | 72 (1) |
| Number of patients seen per week | 0 (0) | 0 (0) | 123 (1) | 3 (0) | *NA* | 196 (2) |
| Number of full-time equivalents in the office | 0 (0) | 5 (0) | 7 (0) | 10 (0) | *NA* | 211 (2) |
|  |  |  |  |  |  |  |
| Percentage of consultations by video | 122 (1) | *NA* | *NA* | 0 (0) | *NA* | 0 (0) |
| Use of connected health tools to monitor the health of patients with chronic diseases | 0 (0) | *NA* | 2 (0) | *NA* | *NA* | 199 (2) |
| Use of electronic patient medical records | 0 (0) | *NA* | 1 (0) | *NA* | *NA* | 33 (0) |
| Possibility to communicate electronically patient clinical summaries | 0 (0) | *NA* | 1 (0) | 254 (3) | *NA* | 38 (0) |
| Possibility to communicate electronically diagnostic and laboratory tests | 0 (0) | *NA* | 1 (0) | 237 (2) | *NA* | 38 (0) |
| Possibility to communicate electronically list of medications | 0 (0) | *NA* | 0 (0) | 282 (3) | *NA* | 46 (0) |
| Practice allowing e-mail or web communications with patients | 0 (0) | *NA* | 0 (0) | 93 (1) | *NA* | 36 (0) |
| Practice allowing online appointment taking with patients | 0 (0) | *NA* | 1 (0) | 274 (3) | *NA* | 30 (0) |
| Practice allowing online medical prescriptions renewal | 0 (0) | *NA* | 3 (0) | 71 (1) | *NA* | 33 (0) |
| Practice allowing online lab results acknowledgement by patients | 0 (0) | *NA* | 4 (0) | 165 (2) | *NA* | 48 (1) |
|  |  |  |  |  |  |  |
| Overall, based on your definition of burnout, how would you rate your current level of burnout? | 0 (0) | *NA* | 32 (0) | 0 (0) | *NA* | 33 (0) |
| How stressful is your job? | 0 (0) | *NA* | 12 (0) | 0 (0) | *NA* | 57 (1) |
| Satisfaction with daily workload | 0 (0) | *NA* | 2 (0) | 0 (0) | *NA* | 35 (0) |
| Satisfaction with time spent on administrative work | 0 (0) | *NA* | 2 (0) | 0 (0) | *NA* | 38 (0) |
| Satisfaction with work-life balance | 0 (0) | *NA* | 0 (0) | 0 (0) | *NA* | 51 (1) |

**Supplementary Table S5**: Regression between burnout self-assessment and burnout-related outcomes and digital health score (N = 9526), unadjusted and adjusted for age, gender, and country. OR: odds ratio, CI: confidence interval.

| Outcomes | OR* (95% CI) | p value |
| --- | --- | --- |
| *Unadjusted* |  |  |
| Burnout self-assessment | 0.98 (0.97,1.00) | 0.054 |
| Perceived stress at work | 1.03 (1.01, 1.04) | 0.001 |
| Dissatisfaction with daily workload | 1.08 (1.06, 1.10) | <0.001 |
| Dissatisfaction with time spent on administrative work | 0.98 (0.97, 1.00) | 0.019 |
| Dissatisfaction with work-life balance | 1.00 (0.98, 1.02) | 0.861 |
| *Adjusted* |  |  |
| Burnout self-assessment | 0.99 (0.97, 1.00) | 0.131 |
| Perceived stress at work | 1.02 (1.00, 1.03) | 0.074 |
| Dissatisfaction with daily workload | 1.08 (1.06, 1.10) | <0.001 |
| Dissatisfaction with time spent on administrative work | 0.99 (0.97, 1.00) | 0.169 |
| Dissatisfaction with work-life balance | 1.00 (0.98, 1.02) | 0.705 |

***** A odds ratio greater than 1 indicate a positive relationship between the digital health score (exposure of interest) and the following outcomes: physician burnout, very or extremely stressful job perception, dissatisfaction with daily workload, time spent on administrative work and work-life balance, respectively.

**Supplementary Figure S6**: Flow chart of the participants (N = 9526).

**
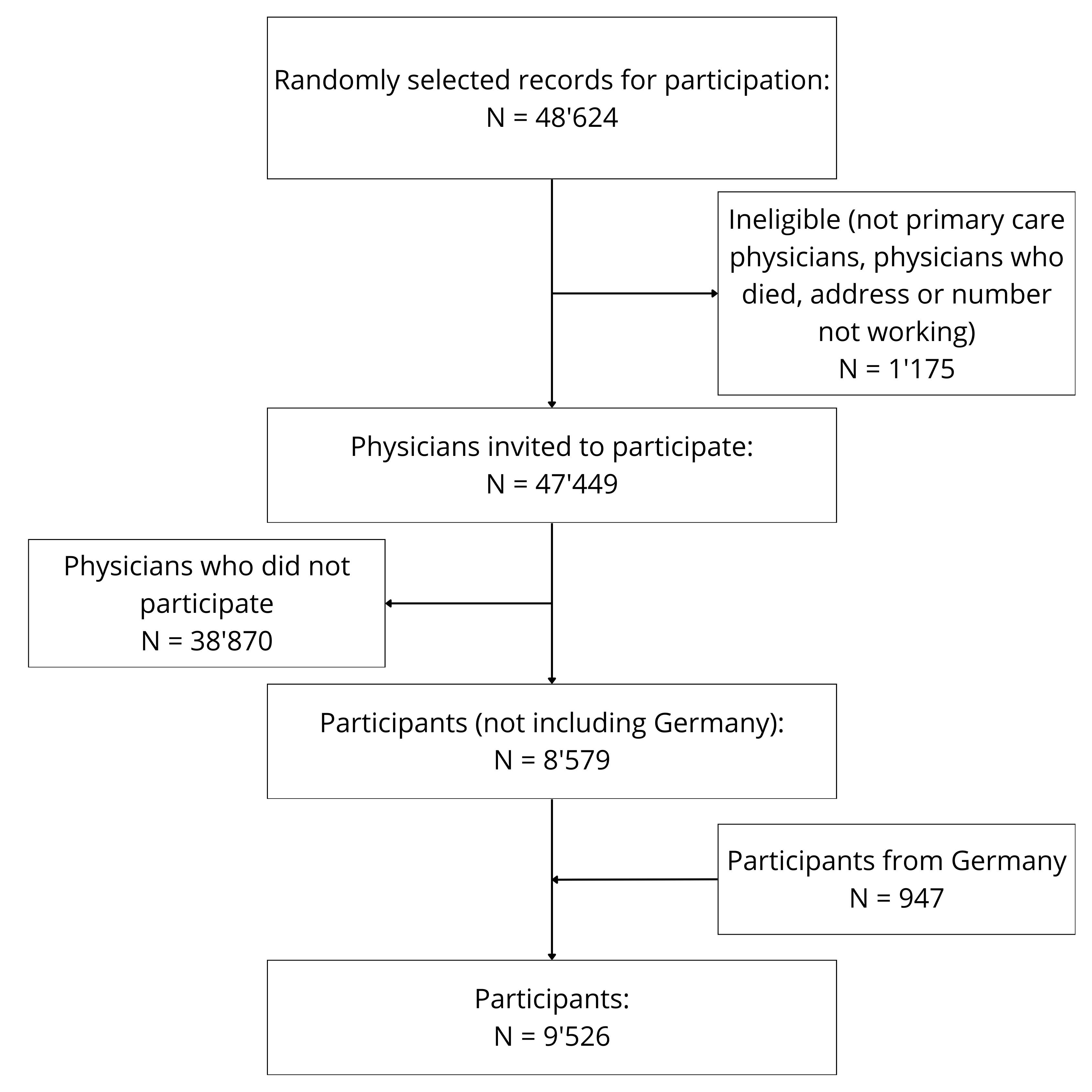
**

**Supplementary Figure S7**: Percentage physician burnout and burnout-related outcomes by country* (N = 9526).

* Country codes: NL = Netherlands, UK = United Kingdom, NZ = New Zealand, SE = Sweden, US = United States, AU = Australia, FR = France, CA = Canada, CH = Switzerland, DE = Germany
